# Promotion of knowledge, attitude, and practice among medical undergraduates regarding infection control measures during COVID-19 pandemic

**DOI:** 10.3389/fpubh.2022.932465

**Published:** 2022-09-15

**Authors:** Abdullah A. Saati, Safa H. Alkalash

**Affiliations:** ^1^Department of Community Medicine and Pilgrims Healthcare, Faculty of Medicine, Umm Al-Qura University, Mecca, Saudi Arabia; ^2^Department of Community Medicine and Healthcare, Faculty of Medicine, Umm Al-Qura University, Al-Qunfudah, Saudi Arabia; ^3^Department of Family Medicine, Faculty of Medicine, Menoufia University, Shibin El-Kom, Egypt

**Keywords:** COVID-19, infection control, knowledge, medical students, skills, training, medical undergraduates

## Abstract

**Background:**

Medical students are exposed to many serious healthcare-associated infections throughout their learning and training time particularly during a pandemic like COVID-19. Therefore, promotion of their knowledge, attitude, and practice regarding infection control measures is a mandatory action.

**Objective:**

This study was executed to evaluate the knowledge, attitude, and practice of medical undergraduates toward infection control measures before and after the implementation of practical infection control training for them.

**Methods:**

A quasi-experimental prospective study was conducted on a simple random sample of medical undergraduates at Al-Qunfudah College of Medicine, Umm Al-Qura University, KSA. A total of 177 medical undergraduates were recruited for this study and completed a predesigned survey on their knowledge regarding infection control measures. Moreover, their practice of infection control measures was evaluated through a checklist before and 6 months after receiving practical infection control training during their academic year 2020–2021.

**Results:**

A total of 177 and 176 responses were obtained at the pre-test and post-test, respectively. The mean ages of students who participated in pre-training and post-training assessments were (22.50 ± 1.02 and 22.03 ± 1.34), respectively with female sex predominance (62.1 and 61.9%). Mean knowledge and practice scores among them were enhanced from (7.79 ± 2.10 and 4.56 ± 2.58) at their pre-test to (11.06 ± 1.27 and 15.68 ± 1.90) at post-test (*P-value* 0.001 and <0.001), respectively. After training, almost all of them recommended infection control training for all medical students due to its great value.

**Conclusion:**

The training course has shown its capability in the promotion of medical undergraduates' knowledge, attitude, and practice toward infection control measures.

## Introduction

Healthcare-associated infections (HCAIs) are common public health problems. They exert increased morbidity and mortality, and increase the health care cost, both in developed and developing countries (1). Between 5 and 10% of patients admitted to modern hospitals in the developed world acquire one or more infections. The risk of healthcare-associated infection in developing countries is doubled many times more than its frequency in developed countries. In some developing countries, the proportion of patients affected by a healthcare-acquired infection can exceed 25% (2).

Hand hygiene plays an important role in controlling and reducing the spread of HAIs but so far it has been observed that practices of hand washing among healthcare personnel are poor (3). The link between hand washing and the spread of disease was established about 200 years ago and until relatively recently (4). The COVID-19 pandemic, which emanated from Wuhan, China, has devastated the global community, disrupting all aspects of human lives (5). Medical students and healthcare workers are more likely to be exposed to SARS-CoV-2 and are, therefore, at a higher risk of COVID-19 infection than other populations in the community (6, 7). However, infection prevention strategies remain the best weapon for protecting healthcare workers against the COVID-19 pandemic (8). Therefore, adherence to infection prevention and control (IPC) guidelines is critical in reducing the risk of coronavirus disease (COVID-19) infection among healthcare workers (9).

Standard precaution is considered to be the most effective measure to limit healthcare-related infections among both patients and healthcare workers and professionals who are strongly advised to stick to these practices. A systematic review reported that adherence to infection control guidelines improves patient safety, prevents healthcare-related infections, and contributes to healthcare workers' safety (10, 11).

Medical students are less knowledgeable than other health care workers (HCWs) about healthcare-associated infections (HAIs) (12). At starting learning in university, a medical student pursuing a degree in health sciences is not required to have accomplished any prerequisites in the area, therefore, his or her undergraduate years are the appropriate time for acquiring the necessary knowledge and skills (13). Training on infection control measures improves the adherence of health care providers to standard precautions (14). For example, teaching infection control measures in medical education is valuable for preventing nosocomial infection and reducing the infection rate (15).

Hence, it is essential for medical students to have an adequate knowledge of infection prevention and control (IPC) practices and to incorporate these into their professional training. Compliance of health care workers and medical students, with standard precautions and hand hygiene has been recognized as an efficient means to prevent and control HCAIs. Such measures not only protect the patient and family but also the HCWs, students, and the environment (16). Therefore, this study focused on elaborating knowledge, attitude, and practice among medical undergraduates regarding infection control guidelines.

## Materials and methods

### Participants

A quasi-experimental prospective study was conducted from October 2020 to November 2021 in the Faculty of Medicine at Al-Qunfudah, Umm Al-Qura University, KSA. The study sample was selected by using a simple random sampling technique. The sample size was calculated using EPI-INFO 7 (17) based on the total number of undergraduates in the Faculty of Medicine at Al-Qunfudah during the academic year (2020–2021), which was 337 students and the frequency of students' knowledge from previous literature was 48.44% so the sample size was 176 medical students at a CI 95% (1). A total of 177 and 176 students completed the pre-and post-intervention tests.

### Study setting

Al-Qunfudah is a Saudi city located on the Red Sea coast near Alieth. Al-Qunfudah College of Medicine was built in 1423H by King Abdullah bin Abdulaziz Al Saud, who was the ex-chair of the Saudi Higher Education Council. It includes nine basic and five clinical departments. It is one of institution in Umm Al-Qura University, one of the largest public universities in KSA, and its main campus is located in Mecca City. It is ranked as 449 in QS World University Rankings 2023.

### Ethical approval

Ethical approval was granted by the University of Umm Al-Qura Ethical Approval Committee to conduct this study, with IRB of UQU reference (ID. HNQI220821). Written consent was obtained from each participant after complete disclosure about the aim and procedure of this study with ensuring anonymity and confidentiality at publishing. Confidentiality was ensured by using an individual online survey and a face-to-face checklist by the two researchers before and after the application of the practical training for medical students, then the results were presented anonymously.

### Tools of data collection

This study made use of an online self-report questionnaire and a predesigned checklist to collect the required data.

A. A predesigned questionnaire was developed by the researchers after revision of previous literature to cover key areas of infection control measures, then it included two sections. The first one; is formed of questions about personal and professional data such as age, gender, academic year, and formal information about infection control. Second section; elicited information about infection control measures including hand hygiene as the duration needed for effective hand hygiene whether using soap and water or alcohol rub also their knowledge about different personal protective equipment.

Scoring of knowledge: each correct response was scored as one (1) point, while an incorrect answer or I do not know was scored as zero (0) points. Every subject who achieved (75% or more) was classified as having good knowledge, (50–75%) was considered fair knowledge, while (<50%) was considered as poor knowledge.

Six directed questions were added to the post-test version to evaluate the participants' attitudes regarding the importance of infection control in health care facilities, the benefits of training, and whether they would recommend it to other medical students.

B. A checklist to determine students' practice of infection control measures which is subdivided into three sections. The first section; involved ten steps of correct hand hygiene. The second section; included five steps for putting on personal protective equipment and the third section; included five steps for putting off personal protective equipment.

Scoring of practices: each assessed practice item was described as “done” which scored (1) point or “not done” which scored (0) point. Subjects who achieved (75% or more) were considered to have a good practice level, while those who obtained (50 to 75%) were considered to have fair practice, and those who scored (<50%) were considered poor in practice.

To confirm the stability of scores over a short period, the test–re-test technique was used. Internal consistency was measured to recognize the level to which the items of the used questionnaire measure the same models and the level to which their items are connected to each other. Internal steadiness assessed reliability by Cronbach's Alpha coefficient test was (0.82).

A pilot study was done on 10% of the study sample (18 medical undergraduates). They were particular from the same location to estimate the clarity and applicability of the study tools, divide approximately the required time for data collection, and recognize the obstacles that may face data collection, and possible actions to overcome. In the light of the obtained data, desirable alterations were done, some questions were complemented and others were explained or omitted.

### Training course

The content of the infection control training course was prepared by both researchers who have experience in infection control and occupational health and is based on the WHO guide for infection control. It was planned to deal with three main domains related to infection control, and each domain consisted of many items: epidemiology of healthcare-associated infections, effective hand hygiene that involved many sub-items such as the importance of effective hand hygiene, five moments for hand hygiene, both types of hand hygiene whether using soap and water or alcohol-based antiseptic solution, finally information regarding different types of PPEs, their uses and the correct sequence of putting on /off PPEs.

### Study procedure

**Figure 1:**
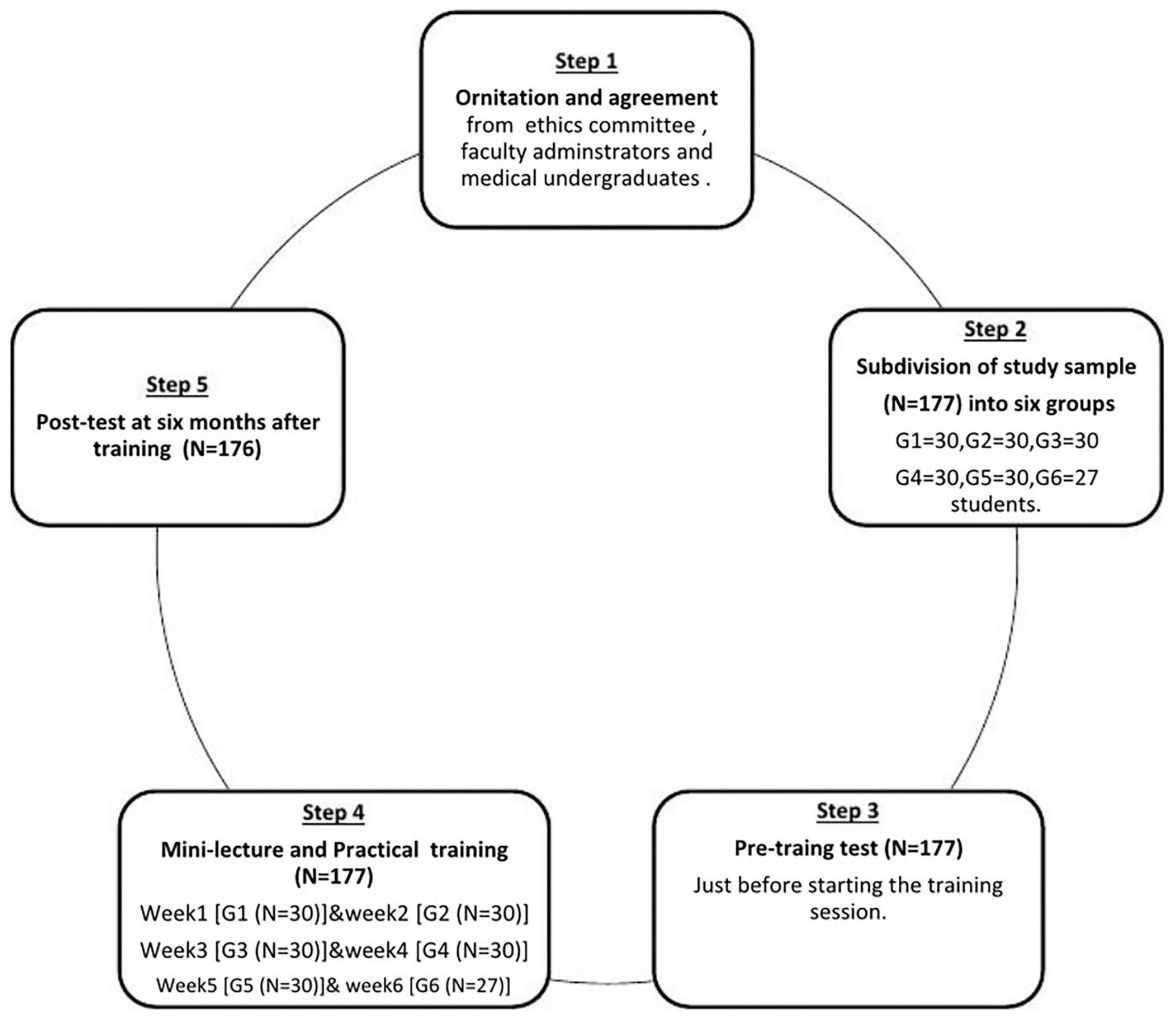
A framework of the study phases scheme.

The study participants were subdivided into six groups in different sessions; each group was formed of about thirty medical undergraduates. The study passed in three phases; pre-intervention, intervention, and post-intervention phases (Figure 1).

Pre-intervention phase; they were invited to submit their responses to an online survey which was sent to all of them through the WhatsApp application to detect their pre-training knowledge regarding infection control measures. Then, after receiving all their responses, they were subjected to an individual assessment for their practice of hand hygiene and putting on/off PPEs.

In an intervention phase; the training course was held starting with a mini-lecture followed by a practical training session where the trainer explained all the steps of effective hand hygiene followed by its demonstration for the trainees. Then they were invited to perform it practically in a group and finally each student had to perform all the correct steps individually and received a friendly and informative feedback about their performance. The same technique was followed in training them to put on/off PPEs.

This approach was repeated for each group separately. Each training session was held once weekly for 6 consecutive weeks.

Post-intervention phase: After about 6 months, the previously trained students were invited to re-check their knowledge, attitude, and practice toward basics of infection control using the same tools and procedure.

### Statistical analysis

The gathered data were coded and analyzed using the Statistical Package for Social Science (SPSS Inc., Chicago, version 22). Quantitative variables were described in the form of mean ± SD. Qualitative variables were expressed as numbers and percentages. Qualitative variables were compared by using Squared Chi or Fisher's exact when the expected frequency is <5. Paired *t-test* was used to compare normally distributed quantitative variables. *P* value less than 0.05 is considered significant.

## Results

**Figure 2:**
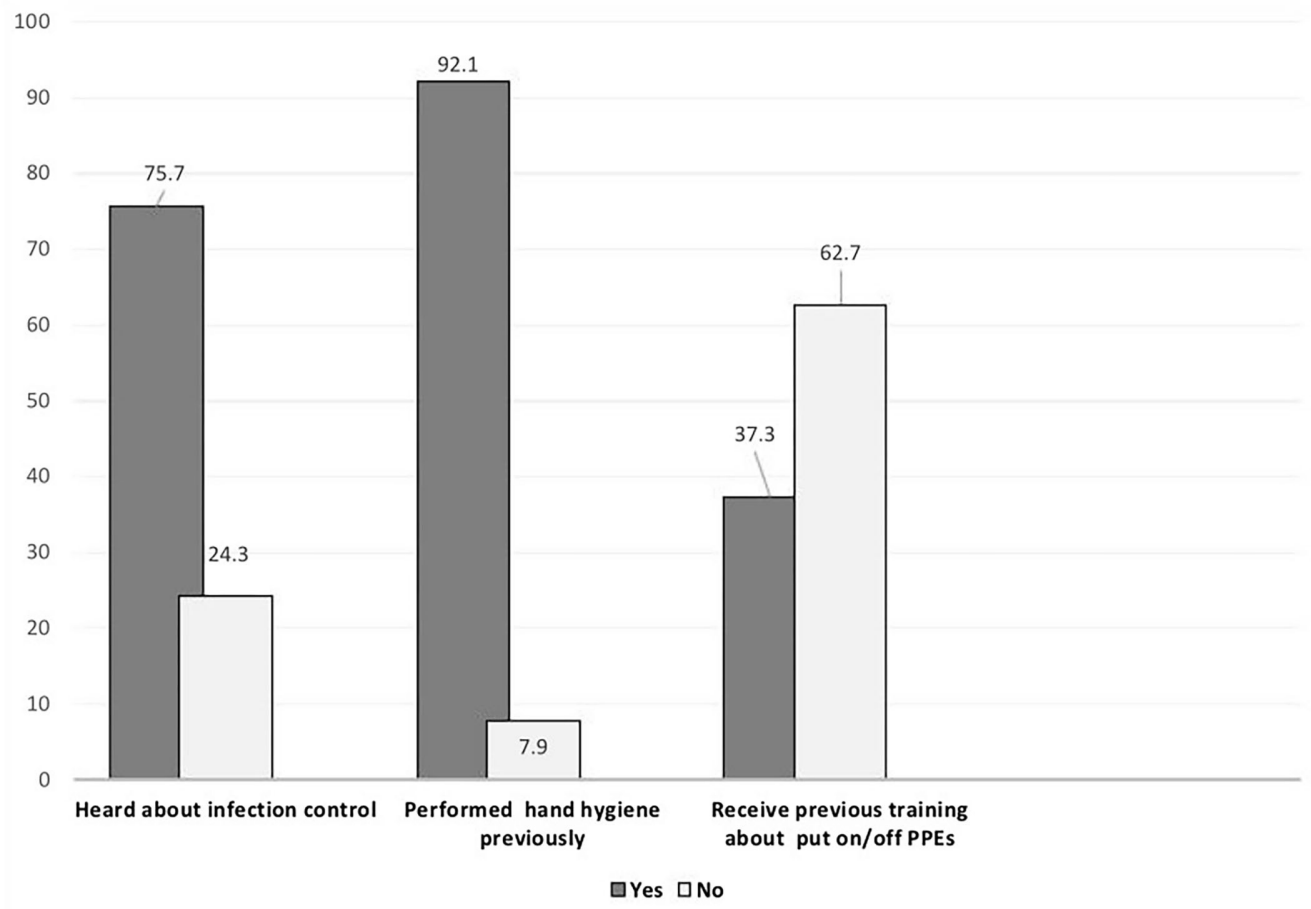
Previous background of medical undergraduates about basic of infection control.

A total of 177 and 176 responses were received at the pre-test and post-test, respectively. The participants' ages ranged (20–25 years) with female sex predominance representing about two-third of the sample (62.1 and 61.9%) at pre-test and post-test, respectively. The majority were in their fourth academic year (Table 1). Although most of them had previous backgrounds in the basics of infection control and hand hygiene (75.0 and 90.0%), respectively, the majority (62.7%) did not receive any practical training regarding the method to put on/off personal protective equipment (PPEs) (Figure 2).

**Table 1:** Demographic data of the participants before and after infection control training.

**Item**	**Pre-training**	**Post-training**	**Test**	***P*-value**
	***N* = 177 (%)**	***N* = 176 (%)**	
Age (M ± SD) (Min.-Max.)	22.50 ± 1.02 (20-25)	22.03 ± 1.34 (20-25)	3.9^**^	0.001^*^
**Gender**				
- Male	67 (37.9)	67 (38.1)	0.002^***^	0.97
- Female	110 (62.1)	109 (61.9)		
**Academic grade**				
- Fourth	79 (44.6)	75 (42.6)	0.53^***^	0.77
- Fifth	53 (29.9)	59 (33.5)		
- Sixth	45 (25.4)	42 (23.9)		

^*^Statistically significant P value < 0.05. ^**^T test; ^***^Chi square test.

In post-test; 80% of them recorded that hand hygiene is the most important item of infection control measures compared to 36% in pre-test setting (*P-*value <0.001). The majority of the students knew accurately in their post-test that the minimum time needed for effective hand hygiene using soap and water is 40 seconds and for alcohol rub 20 s. (96.6 and 88.1%) in comparison to (54.0 and 56.0%) in their pre-test (*P-*value < 0.001). About 92.0% of them had great improvement in their knowledge as regards five moments for hand hygiene (*P-*value < 0.001). Almost all of them ensured the necessity to remove jewelries or accessories and completely bare hands before starting hand hygiene and all of them were able to identify different types of PPEs (*P-*value < 0.001). About 90.0 and 93.0% of them reported that hand hygiene is the first and last step to putting on/off PPEs compared to 66.0 and 64.0% before receiving training (*P-*value < 0.001). Regarding the correct sequence of putting on PPEs; most of them (93.0 and 77.0%) reported that gown were the first PPEs to be worn while gloves were the last to be put off in post-test, which was greater than the pre-test frequency (41.0 and 20.0%) (*P-*value <0.001) (Table 2).

**Table 2:** Comparison between students' knowledge about infection control measures before and after training.

**Items**	**Pre-test**	**Post-test**	**X^2^**	***P*-value**
	***N* = 177 %**	***N* = 176 %**		
**The most important component of infection control measures**				
Hand hygiene#	64 36.2	141 80.2	70.57	<0.001^*^
PPEs	97 54.8	32 18.1		
Cleaning surface	16 9.0	3 1.7		
**Hand hygiene embedded in your professional practice**				
No	15 9.6	2 1.1	10.37	0.001^*^
Yes	162 90.4	174 98.9		
**Minimum time needed for hand washing using soap and water**				
Incorrect	82 46.3	6 3.4	86.86	<0.001^*^
Correct answer (40 s)	95 53.7	170 96.6		
**Minimum time needed for hand hygiene using Alcohol rub**				
Incorrect	78 4.1	21 11.9	45.16	<0.001^*^
Correct answer (20 s)	99 55.9	155 88.1		
**Know five moments for hand hygiene**				
No	77 43.5	2 1.1	91.19	<0.001^*^
Yes	100 56.5	174 98.9		
**One of five moments for hand hygiene**				
Incorrect	65 36.7	13 7.4	44.12	<0.001^*^
Correct answer (after touching a patient)	112 63.3	163 92.6		
**Steps for proper hand hygiene**				
Incorrect	143 80.8	60 34.1	78.76	<0.001^*^
Correct answer (ten steps to rub hands properly)	34 19.2	116 65.9		
**It is obligatory to remove any jewelries/accessories before starting hand hygiene**				
No	10 5.6	0 0	Fischer exact	0.002^*^
Yes	167 94.4	176 100		
**Know about personal protective equipment**				
No	37 5.6	0 0	Fischer	<0.001^*^
Yes	140 94.4	176 100	exact	
**The first step for putting on personal protective equipment**				
Hand hygiene#	118	66.7 159	90.3	29.28	<0.001^*^
Wearing of gown.	29 16.4	7 4.0		
Putting on face mask	12 6.8	7 4.0		
Putting on gloves	18 10.2	3 1.7		
**The first personal protective equipment to put on**				
Gown#	72 40.7	164 93.2	120.53	<0.001^*^
Mask	24 13.6	6 3.4		
Goggles	79 44.6	2 1.1		
Gloves	2 1.1	4 2.3		
**The last personal protective equipment to put off**				
Gloves#	36 20.3	136 77.3	124.31	<0.001^*^
Gown	72 40.7	17 9.7		
Mask	44 24.9	5 0.6		
Goggles	25 14.1	18 12.5		
**The step after putting off personal protective equipment PPEs**				
Collect disposal	48 27.1	11 6.3	43.15	<0.001^*^
Hand hygiene#	113 63.8	163 92.6		
Leaving patient room	16 9.0	2 1.1		

* Statistically significant P-value < 0.05 # Correct answer.

Good levels of knowledge and practice were improved after training represented 71.0% and 55.7% of the study sample respectively (Table 3). Mean knowledge scores among medical students increased from 7.79 ± 2.10 at pre-test to 11.06 ± 1.27 at post-test and the same was observed regarding their practice which significantly improved from 4.56 ± 2.58 at pre-test to 15.68 ± 1.90 at post-test (*P*-values 0.001 and <0.001), respectively (Table 4).

**Table 3:** Comparison between participants' mean knowledge and practice scores of infection control measures before and after training.

**Item**	**Pre-training**	**Post-training**	***P*-value of *X ^2^* test**
	***N* = 177 (%)**	***N* = 176 (%)**	
**Total score of knowledge**			
Good	15 (8.5)	125 (71.0)	0.0001^*^
Fair	82 (46.3)	49 (27.9)	
Poor	80 (45.2)	2 (1.1)	
**Total score of practices**			
Good	0 (0.0)	98 (55.7)	0.0001^*^
Fair	7 (4.0)	78 (44.3)	
Poor	170 (96.0)	0 (0.0)	

* Statistically significant P-value < 0.05.

**Table 4:** Distribution of studied participants according to total knowledge and practices scores of infection control measures before and after intervention.

**Item**	**Pre-training (M ±SD)**	**Post-training (M ±SD)**	**Paired *T*-test *P-*value**
	***N* = 177**	***N* = 176**	
Knowledge about hand hygiene	5.1 ± 1.46	6.59 ± 0.72	0.00001^*^
Knowledge about PPEs	2.64 ± 1.22	4.47 ± 0.79	0.0001^*^
Total knowledge score	7.79 ± 2.10	11.06 ± 1.27	0.001^*^
Practice score regarding doing hand hygiene	2.49 ± 1.61	7.10 ± 1.28	0.0001^*^
Practice score regarding putting on PPEs	0.94 ± 1.06	4.31 ± 0.72	0.0001^*^
Practice score regarding putting off PPEs	1.13 ± 1.08	4.27 ± 0.70	0.0001^*^
Total practice score	4.56 ± 2.58	15.68 ± 1.90	0.0001^*^

* Statistically significant P value <0.05.

Regarding their attitude toward infection control measures; almost all of them decided that basic infection control training is beneficial for medical staff health and recommended its application for all medical students due to its health-related benefits (Table 5).

**Table 5:** Students' attitude toward basics of infection control after training.

**Item**	**Frequency (%)**
**Basics infection control training is valuable for health care providers**	
Yes	176 (100.0)
No	0 (0.0)
**Students recommended infection control training for other medical students**	
Yes	176 (100.0)
No	0 (0.0)
**Infection control training is an integral part of health care services**	
Yes	169 (96.0)
No	7 (4.0)
**Health providers should be adherent to infection control measures during dealing with patients**	
Yes	160 (90.9)
No	16 (9.1)
**Health providers could handle body fluids with bare hands when gloves are not available**	
Yes	0 (0.0)
No	176 (100.0)
**Health providers have to wash hands even when they used gloves**	
Yes	163 (92.6)
No	13 (7.4)

## Discussion

This study highlighted the influence of infection control training on the knowledge, attitude, and practice of medical undergraduates toward infection control measures pre and 6 months post-training. The majority of the study sample had previous background regarding infection control measures, specifically hand hygiene while there was a shortage in their knowledge regarding different forms of personnel protective equipment and its uses, which makes these results pass parallel to several studies which reported that combined knowledge, attitude, and practice (KAP) scores on hand and attire hygiene were moderate while equipment hygiene was unsatisfactory (18, 19). These data denote that there was some sort of insufficiency in the availability of clear information about different forms of personnel protective equipment for medical students while hand hygiene was more commonly practiced by them for many reasons; the most important one is the COVID-19 pandemic and its great impact in pushing all individuals to effectively wash their hands. Another cause is that all governmental, and non-governmental health care facilities and social media were keen to provide continuous and focused public training programs for proper hand hygiene during the COVID-19 pandemic. In addition to the incorporation of infection control lectures in their education courses, which made them have fair knowledge of it apart from different types of personnel protective equipment.

The study group did not receive any kind of formal training on infection control measures. Therefore, the baseline knowledge that was obtained during their pretest probably had its link with what was taught to them during lectures and daily routines. As expectedly, students' previous knowledge of the basic infection control routine was unsatisfactory, as was evident from the low percentages of correct pretest answers. However, the percentages of students who gave correct answers significantly improved after the didactic and practical sessions. The same was previously noticed by an Indian study which concluded that their infection control educational intervention, which was provided for a sample of the nursing staff had a significant impact on the improvement of the participants' knowledge (20). This result is also consistent with another study that reported that infection control knowledge among undergraduate nursing students in their 3rd year was improved whatever the method of infection control training (21).

Students' skills in performing effective hand hygiene, putting on and putting off PPEs showed significant improvement after training in comparison to their practice before training, respectively and this achievement is highly appreciated as not only their knowledge about the basics of infection control was improved but also their practice which is the most important target in order to ensure their safety as well as patients' safety. This finding was supported by what was gained by an Egyptian study which revealed that the practice of medical interns for basics of infection control measures was significantly promoted through the application of different modalities of training (role play, case study, case scenarios,..) (22).

Authentic and effective practical training may motivate the trainees to change their attitude toward an important issue like commitment to infection control measures. In this study, medical undergraduates showed a positive attitude and more satisfaction with the applied infection control training to the extent that they recommended its repetition for other medical students due to its great value in saving their lives also their families' and patients' lives. This important finding is similar to that was extracted by a study in Qatar as the authors recommended that multifaceted training programs should target freshly graduated medical students or the training should to be included within their medical curriculum to enable students to adopt and adhere to IPC guidelines and reason beyond this conclusion is that 61.90% were satisfied with their training in infection prevention and control, and 66.13% were trained in effective hand hygiene (1).

### Study limitations

The study had some limitations that should be acknowledged. The quasi-experimental design cannot rule other factors out of control, whereas the study results revealed that the knowledge of the participants was elaborated after receiving infection control training, however, they may read or receive information about infection control measures from any other sources during the period between pre and post-training. This study was conducted among medical students at one site, which is the faculty of medicine at Al-Qunfudah, Umm Al-Qura University, KSA so it could not reflect medical students' knowledge or practice in other universities. Another limitation was related to choosing an appropriate time for the application of infection control training without interfering with their learning schedule, so they were subdivided into six subgroups over 6 weeks and each group received their training session once. Post-training test was another challenge because students were busy and about to undergo their final exam, so the researchers notified them to have their post-test whenever possible.

### Study strengthens

One of the most important strengths of this study was the actual training of medical students to wash their hands, put on/off PPEs in the proper way, and make them practice it well-under the observation of a qualified trainer. This on-site training, evaluation, and re-evaluation ensure that medical students will practice it professionally. Medical students' conviction of the importance to following the basics of infection control will increase the community's commitment to infection control methods because these students are considered as ambassadors to educate the public on all measures to prevent and control the spread of diseases, especially during the period of the COVID-19 pandemic.

## Conclusion

The infection control training was effective in promoting students' knowledge, attitude, and practice of infection control measures. Medical undergraduates recognized the worth of commitment to infection control measures and recommended training applications for other medical students due to their role in saving lives. Based on the study findings; we recommend frequent and regular training for all medical students on infection control measures and incorporate this training course as a fundamental part of their longitudinal educational courses. Medical students are our ambassadors for public health education and training, so when they understand well and accept the commitment to infection control measures, they will be able to expand this important knowledge and skills among the public.

## Data availability statement

The raw data supporting the conclusions of this article will be made available by the authors, without undue reservation.

## Ethics statement

The studies involving human participants were reviewed and approved by University of Umm Al-Qura Ethical Approval Committee. The patients/participants provided their written informed consent to participate in this study.

## Author contributions

AS: conceptualization and designed study, designing instruments of data collection, research materials, and organized data, wrote the initial and final draft of an article, editing, reviewing, article, and provided logistic support. SA: conceptualization and designed study, designing instruments of data collection, provided research materials, implemented the training, collected, and organized data, data analysis and interpretation, editing, reviewing, drafting article and revising it critically for important intellectual content, and finally proofed and submitted it on the journal web site. Both authors contributed to the article and approved the submitted version.

## Conflict of interest

The authors declare that the research was conducted in the absence of any commercial or financial relationships that could be construed as a potential conflict of interest.

## Publisher's note

All claims expressed in this article are solely those of the authors and do not necessarily represent those of their affiliated organizations, or those of the publisher, the editors and the reviewers. Any product that may be evaluated in this article, or claim that may be made by its manufacturer, is not guaranteed or endorsed by the publisher.
