# Supplementary material for: Promotion of knowledge, attitude, and practice among medical undergraduates regarding infection control measures during COVID-19 pandemic
Source: Front Public Health. 2022 Sep 15;10:932465. doi: 10.3389/fpubh.2022.932465 (PMC9521352; doi:10.3389/fpubh.2022.932465)
Supplement: Supplementary file 1 [file Data_Sheet_1.PDF]

<u>Checklist to assess students' practice of infection control measures (CDC, 2020)</u>

Procedure	Done	Not done
**Hand hygiene**		
1. Remove jewelries and bare hands.		
2. Put adequate amount of antiseptic solution.		
3. Rub hands palm to palm.		
4. Rub back of each hand with palm of other hand with fingers interlaced.		
5. Rub hands with fingers interlaced.		
6. Rub with back of fingers to opposing palms with fingers interlocked**.**		
7. Rub each thumb clasped in opposite hand using a rotational movement**.**		
8. Rub tips of fingers in opposite palm in a circular motion**.**		
9. Rub each wrist with opposite hand.		
10. Rinse hands thoroughly with tepid running water.		
11. Use elbow to turn off tap.		
12. Dry thoroughly with single-use towel.		
**Wearing PPEs**		
1. Identify and gather the proper PPE to don.		
2. Perform hand hygiene using hand sanitizer.		
3. Put on isolation gown.		
4. Put on a facemask.		
5. Put on face shield or goggles.		
6. Put on gloves.		
**Taking Off (Doff) PPE**		
1. Remove gloves		
2. Remove gown		
3. Perform hand hygiene		
4. Remove face shield or goggles		
5. Remove and discard facemask		
6. Perform hand hygiene		
