# Supplementary material for: Promotion of knowledge, attitude, and practice among medical undergraduates regarding infection control measures during COVID-19 pandemic
Source: Front Public Health. 2022 Sep 15;10:932465. doi: 10.3389/fpubh.2022.932465 (PMC9521352; doi:10.3389/fpubh.2022.932465)
Supplement: Supplementary file 2 [file Data_Sheet_2.PDF]

Students' knowledge and attitude towards infection control measures (Pre and post-training)

1. Name 2.Age 3. Gender 4. Academic year

4. Do you know basics of infection control measures?

A) Yes B) No

5. What's the most important method to prevent spread of infection in hospital environment?

A. Proper hand hygiene.
B. Proper wearing of personal protective equipment.
C. Proper cleaning of surfaces.

6. Is hand hygiene embedded in your professional practice?

A) Yes B) No

7. What's minimum time needed for hand washing using soap and water?

A. 60-70 sec. B.40-60 sec. C. 30 -40 sec. D. 10-20 sec.

8. What's minimum time needed for hand hygiene using Alcohol rub?

A. 60-70 sec. B.40-60 sec. C. 30 -40 sec. D. 10-20 sec.

9. Do you know five moments for hand hygiene?

A) Yes B) No

10. Which one of the following belongs to five moments for hand hygiene?

A) Before entering the day clinic.
B) After touching a patient.
C) Before leaving bathroom.

11. What is number of steps to rub hands during proper hand hygiene?

A. Seven steps B. Eight steps C. Nine steps D. ten steps

12. Is it obligatory to remove any jewelries before starting hand hygiene?

A) Yes B) No

13. Do you know types of personal protective equipment?

A) Yes B) No

14. What's the first step for putting on personal protective equipment?

A. Hand hygiene.

B. Wearing of gown.

C. Putting on face mask.

D. Putting on gloves.

15. What's the first personal protective equipment to put on?

A. Gown B. Face mask C. Goggles D. Gloves

16. What's the last personal protective equipment to put off?

A. Gloves B. Gown C. Face mask D. Goggles

17. What's the step after putting off personal protective equipment PPEs?

A. Collecting the disposed PPEs.
B. Hand hygiene.
C. Leaving patient room.

18. Is the training on basics of infection control beneficial for healthcare providers?

A. Yes
B. No

19. Do you recommend training of other medical students on basics of infection control?

A. Yes
B. No

20. Do you perceive that infection control training is an integral part of health care services?

A. Yes
B. No

21. **Do you think that is necessary for health providers to be adherent to infection control measures during dealing with the patients?**

A. Yes
B. No

22. Could health care providers handle body fluids with bare hands when gloves are not available?

A. Yes
B. No

23. Should healthcare providers wash hands even when they used gloves?

A. Yes
B. No
